# Synergistic effects of seed disperser and predator loss on recruitment success and long-term consequences for carbon stocks in tropical rainforests

**DOI:** 10.1038/s41598-017-08222-4

**Published:** 2017-08-09

**Authors:** Laurence Culot, Carolina Bello, João Luis Ferreira Batista, Hilton Thadeu Zarate do Couto, Mauro Galetti

**Affiliations:** 1 Universidade Estadual Paulista (UNESP), Instituto de Biociências, Departamento de Zoologia, Laboratório de Primatologia, Avenida 24A, 1515, 13506-900, CP199 Rio Claro, SP Brazil; 2 Universidade Estadual Paulista (UNESP), Instituto de Biociências, Departamento de Ecologia, Laboratório de Biologia da Conservação, Avenida 24A, 1515, 13506-900, CP199 Rio Claro, SP Brazil; 3 Escola Superior de Agricultura “Luiz de Queiroz” (ESALQ) / Universidade de São Paulo (USP), Departamento de Ciências Florestais, Avenida Pádua Dias, 11, 13418-900 Piracicaba, SP Brazil

## Abstract

The extinction of large frugivores has consequences for the recruitment of large-seeded plants with potential lasting effects on carbon storage in tropical rainforests. However, previous studies relating frugivore defaunation to changes in carbon storage ignore potential compensation by redundant frugivores and the effects of seed predators on plant recruitment. Based on empirical data of the recruitment success of a large-seeded hardwood tree species (*Cryptocarya mandioccana*, Lauraceae) across a defaunation gradient of seed dispersers and predators, we show that defaunation increases both seed dispersal limitation and seed predation. Depending on the level of seed predator loss, plant recruitment is reduced by 70.7–94.9% as a result of the loss of seed dispersers. The loss of large seed predators increases the net seed mortality by 7–30% due to the increased abundance of small granivorous rodents. The loss of large seed dispersers can be buffered by the compensatory effects of smaller frugivores in seed removal, but it is not sufficient to prevent a decrease in plant recruitment. We show that the conservation of both seed predators and dispersers is necessary for the recruitment of large-seeded plants. Since these plants contribute substantially to carbon stocks, defaunation can jeopardize the maintenance of tropical forest carbon storage.

## Introduction

Anthropocene defaunation, known as the local or global extinction of animal populations or species, is recognized as an important driver of global environmental change^1^. Indeed, defaunation extends well beyond species loss; it concerns a shift in species composition and its impact on ecological and evolutionary processes and on ecosystem services^2–5^. Previous studies highlight the impact of defaunation on ecological services such as pollination, seed dispersal, nutrient cycling and decomposition, water quality, and dung removal^1, 6, 7^ and, more recently, on carbon storage^8–10^. Despite the evidence suggesting that the decline or loss of frugivores affects plant recruitment success and leads to changes in plant communities^11^, the estimation of the magnitude of the effects of defaunation on future carbon storage has been based on inferences that frugivore extinction will necessarily lead to direct effects on plant species fitness^8–10^. However, in defaunated scenarios, a decrease in seed predation pressure may buffer the effects of seed disperser loss^12^ and many plant species can still recruit without or with few dispersers^13^ or have multiple dispersers that could buffer plant extinction^14, 15^.

Therefore, the effects of frugivores on carbon storage have been based on simple models that do not consider the potential consequences of the altered seed predator community. It is well known that plant recruitment depends on the activities of both mutualists (seed dispersers) and antagonists (seed predators, herbivores)^16^. Vertebrate defaunation leads to significant changes in the communities of both seed dispersers and predators and, while some species respond negatively to anthropogenic activities, others can benefit because of differential functional response traits or competitive (numerical) release^12^. This demographic asynchrony can give rise to compensatory effects^17^ that are able to mitigate, fully compensate or even invert the effects of defaunation on the seed dispersal process^18^. Compensatory effects are possible if a certain degree of redundancy exists in seed dispersal and predation services^14, 19^. Therefore, functional redundancy among mutualist and antagonist species and possible compensatory effects still need to be investigated in a defaunation context.

While the traditional determination of changes in seedling communities highlights important defaunation effects on the future plant composition of tropical forests^20, 21^, they do not enable the identification of the underlying processes leading to this result. The detailed study of one plant species likely to be affected by defaunation enables to understand and disentangle the effects of mutualistic and antagonistic interactions, identify the causes of recruitment failure, and highlight the mechanisms underlying possible compensatory effects of the resilient frugivore community^22^. Here, we address the effects of defaunation, of both seed dispersers and predators, on the recruitment success of a large-seeded hardwood tree. We investigated the contributions of the seed dispersers (southern muriquis – *Brachyteles arachnoides*, southern brown howler monkeys – *Alouatta guariba*, and black-fronted piping guans, hereafter called jacutingas – *Aburria jacutinga*) of a large-seeded hardwood tree species, *Cryptocarya mandioccana* (Lauraceae), in three areas across a defaunation gradient of seed dispersers and predators to assess the magnitude of possible compensatory effects. The seed dispersers and the seed predators (peccaries – *Pecari tajacu* and *Tayassu pecari*, agoutis – *Dasyprocta* sp., and small rodents) range from large (220 kg) to small (0.01 kg) in size (Table 1), and they respond to defaunation according to their body size (from the largest to smallest frugivore). By comparing seed dispersal effectiveness among the seed dispersers, we predicted the relative impacts of their local extinction on plant recruitment, taking into account possible compensatory effects and changes in the seed predator community.

**Table 1:** Seed disperser and predator assemblages in the intact (Carlos Botelho, highlands – CB-High), moderately defaunated (Ilha do Cardoso – IC), and defaunated areas (Carlos Botelho, lowlands – CB-Low).

Functional group	Species	Common names	Mass (kg)	CB - High (Non-def)	IC (Mod def)	CB - Low (Def)
Seed dispersers	*Tapirus terrestris*	Tapir	220	com	ex	fe
	*Brachyteles arachnoides*	Muriqui	12	com		fe
	*Alouatta guariba*	Howler monkey	8	com	com	fe
	*Aburria jacutinga*	Jacutinga	1.2	com	com	com
Seed predators	*Tayassu pecari*	White-lipped peccary	35	fe	com	low
	*Pecari tajacu*	Collared peccary	15	com	com	com
	*Cuniculus paca*	Spotted Paca	5	com	com	com
	*Dasyprocta leporina*	Red-rumped Agouti	3	low	com	com
	*Trinomys iheringi*	Ihering´s Spiny Rat	0.4	com	com	com
	*Euryoryzomys russatus*	Russet Rice Rat	0.2	com	com	com
	*Juliomys pictipes*	Lesser Wilfred’s Mouse	0.02	com		com
	*Sooretamys angouya*	Paraguayan rice rat	0.02	com		com
	*Thaptomys nigrita*	Blackish Grass Mouse	0.01	com		com
	*Oligoryzomys nigripes*	Black-footed Pygmy Rice Rat	0.01	com	com	com
	*Akodon montensis*	Montane Grass Mouse	0.01	com		com

‘com’ indicates that the species is common in the area, “low” that it occurs in low density, “fe” that the species is functionally extinct, and “ex” that the species is extinct.

## Results

### Contribution of seed dispersers to recruitment success

**Figure 1:**
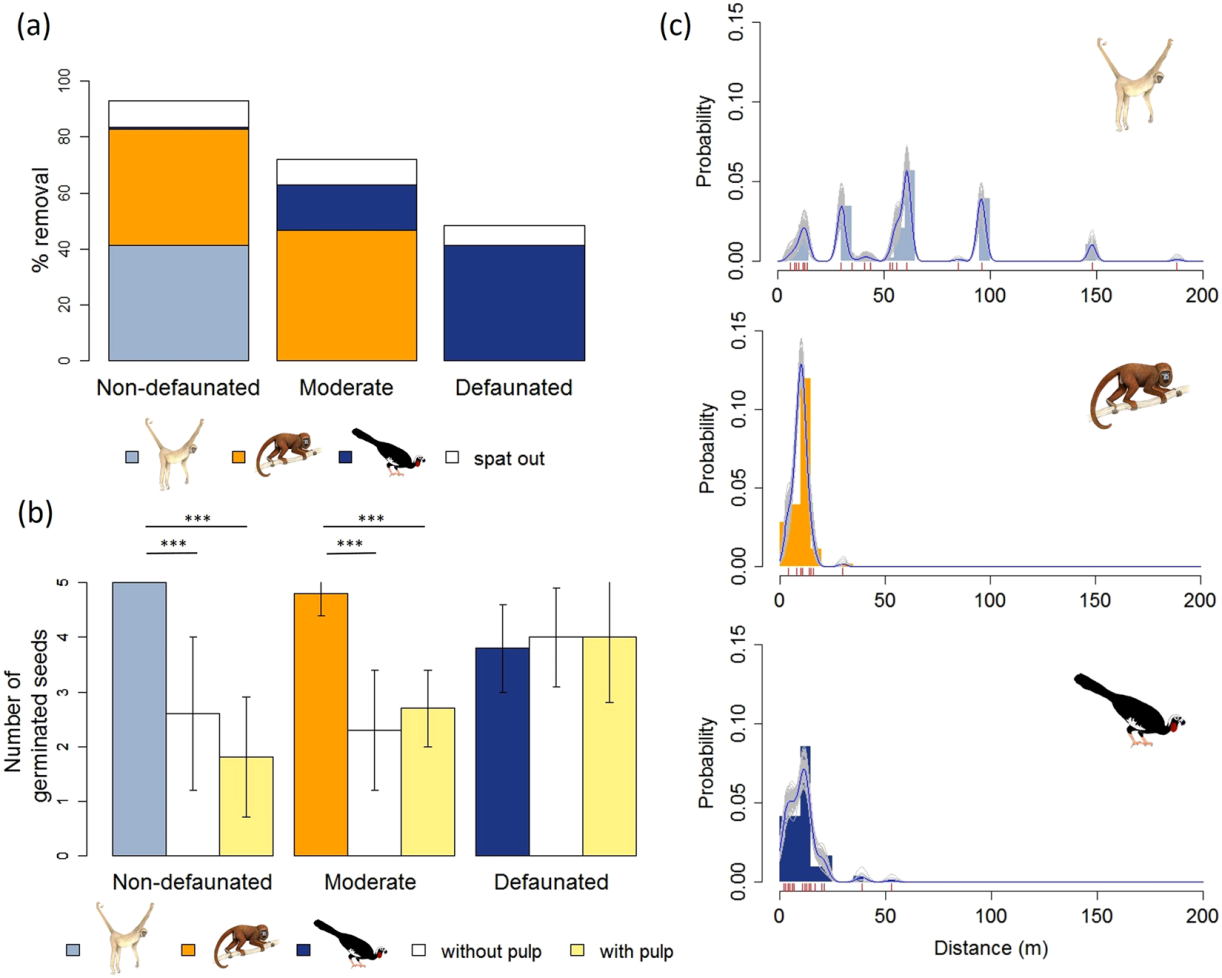
Components of seed dispersal effectiveness of the three main dispersers of *C. mandioccana*. (**a**) Percentage of seeds swallowed by muriquis, howler monkeys, and jacutingas, as well as the percentage of spat out seeds (all frugivore combined) in the three study sites characterized by different seed disperser communities: Non defaunated (muriquis, howler monkeys, and jacutingas), Moderate (howler monkeys and jacutingas), and Defaunated (jacutingas). Data are based on focal observations of *C. mandioccana* fruiting trees in 2011–2012 (Non-defaunated: N = 172 h; Moderate: N = 108 h, and Defaunated: N = 153 h). (**b**) Germination success of seeds defecated by muriquis, howler monkeys, and jacutingas, compared to seeds without pulp and seeds with pulp along the defaunation gradient. Bars represent the mean number of germinated seeds out of five seeds (ten replicates) and vertical lines represent standard deviation. (**c**) Seed dispersal distances to *C. mandioccana* conspecifics by muriquis (N = 173), howler monkeys (N = 127), and jacutingas (N = 168). Frequency distributions of seed dispersal distances (5 m-bins) where red vertical bars represent each observed dispersal event and the blue and grey lines, a non-parametric smoothing spline fit to the empirical distance distributions together with bootstrapped estimates. (Illustrations of: muriqui and howler monkey - Copyright Stephen D. Nash; jacutinga – Copyright Fabio Martins Labecca, authorized by the authors).

We estimated recruitment success, and the contribution of each seed disperser to the recruitment success, of *C. mandioccana* in three areas of Atlantic Forest differing in their seed disperser and predator communities (Table 1; see Fig. S1 in Supplementary Information). The lowest recruitment success was in the intact forest (9%), while it was highest in the moderately defaunated forest (15.51%). The most defaunated forest presented an intermediate value (12.77%) (see Table S2). The quantity and quality components of seed dispersal effectiveness (Fig. 1) as well as of seed predation (see Table S2 and Fig. S3) explain these results. The overall contribution of jacutingas and howler monkeys increased along the seed disperser defaunation gradient (see Table S2). Jacutingas contributed only 0.7% towards *C. mandioccana* recruitment success where they occur together with larger-bodied primates (muriquis and howler monkeys), while their contribution reached 61.4% where they are the only seed disperser (see Table S2). This pattern is mostly explained by seed removal (Fig. 1a). Indeed, there is a partial compensatory effect in seed removal with an increasing contribution of the remaining seed dispersers, such as howler monkeys (from 41 to 47%) and jacutingas (from 1% to 16% and then to 41%), along the defaunation gradient (Fig. 1a, see Table S2). Despite this functional compensatory effect, we observed a decreasing proportion of swallowed seeds, i.e., seeds dispersed away from the parent tree, with the loss of seed dispersers: 83% with the complete assemblage, 63% without muriquis, and 41% without muriquis and howler monkeys (Fig. 1a).

The germination success of seeds defecated by muriquis and howler monkeys was significantly higher than that of non-defecated seeds with or without pulp (muriquis: N = 10, F = 24.1, P < 0.0001; howler monkeys: N = 10, F = 30.8, P < 0.001; Fig. 1b). Conversely, the germination success of seeds defecated by jacutingas did not significantly differ from that of non-defecated seeds with or without pulp (N = 9, F = 0.1, P = 0.9) (Fig. 1b; see Supplementary Method S4). Muriquis, a large-bodied frugivore, dispersed seeds about six times farther than smaller-bodied howler monkeys and jacutingas (N = 468, F = 271.26, P < 0.05; post hoc test: P < 0.0001), with a mean of 59.7 ± 35.7 m from the nearest conspecific compared to 9.7 ± 3.4 m and 10.5 ± 7.3 m for howler monkeys and jacutingas, respectively (Fig. 1c). However, while muriquis largely contributed, quantitatively, to recruitment success through high seed removal (Fig. 1a), their contribution was qualitatively low due to extremely high seed mortality at all distances at the site where they occur (see Table S2 and Fig. S3). Survival tended to increase with distance but this effect was only significant in the moderately defaunated site in 2011 (N = 240, Z = 2.01, P < 0.05) and in the defaunated site in 2012 (N = 240, Z = 2.17, P < 0.05) (see Fig. S3), with both sites harboring a more complete assemblage of seed predators.

### Compensatory effect and expected recruitment success

**Figure 2:**
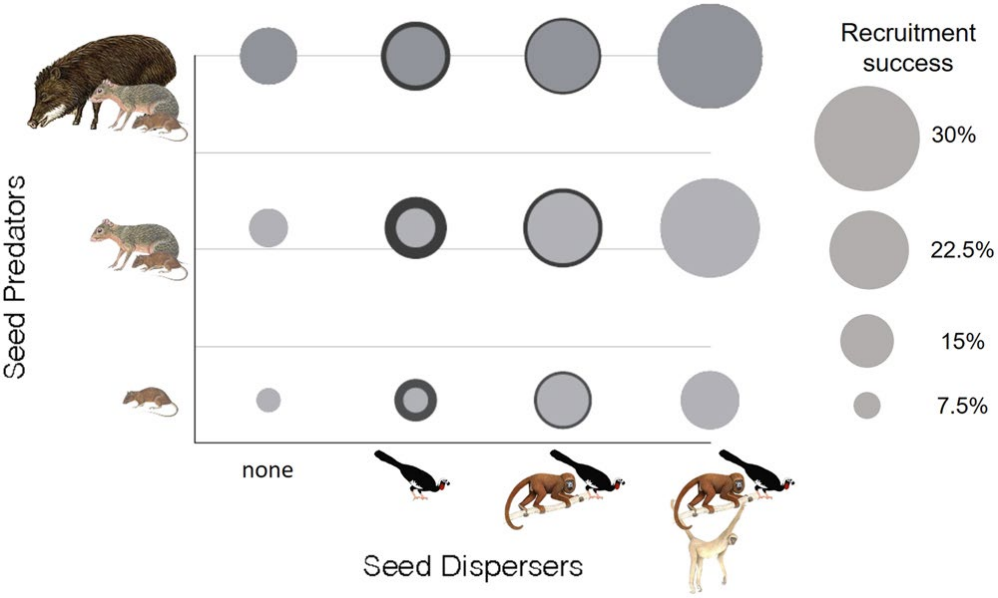
Expected recruitment success of *C. mandioccana* with and without compensation effect of the disperser community. The loss of seed dispersers was simulated in the area where the complete assemblage of seed dispersers is currently present (Carlos Botelho State Park, highlands) and the recruitment success of *C. mandioccana* estimated in three scenarios of seed predator communities. The simulations give the expected recruitment success in different scenarios of seed disperser (x axis) and seed predator (y axis) communities considering (dark grey circles) or not (light grey circles) compensation effects of the disperser community. The contribution of the disperser community in the compensation scenario is based on the data collected in Cardoso Island (community without muriquis) and in the lowland part of Carlos Botelho State Park (community without muriquis and howler monkeys). (Illustrations of: muriqui and howler monkey - Copyright Stephen D. Nash; peccary, agouti, and rodent – Copyright Fiona A. Reid; jacutinga – Copyright Fabio Martins Labecca, authorized by the authors).

We simulated a sequence of seed disperser loss (from the largest to smallest frugivore) in the intact area to explore how seed disperser and predator extinctions could affect *C. mandioccana* recruitment. We took into account three scenarios based on the seed predator community and compensatory effects of the disperser community. In all scenarios of seed disperser loss, recruitment success decreased with the decline in the richness of seed predators (Fig. 2), possibly because of increased predation pressure by small rodents as evidenced by the higher frequency of visits by small rodents to *C. mandioccana* fruits in the seed predator-defaunated site (see Table S5). Taking the recruitment success of the scenario with the most intact seed predator community as reference, the loss of all seed dispersers would decrease the recruitment success of *C. mandioccana* individuals by 70.7% if the complete set of seed predators is maintained, by 86.7% if coupled with the extinction of peccaries, and by 94.9% if coupled with the extinction of both peccaries and agoutis (Fig. 2).

Taking the recruitment success of each seed predation scenario as a reference, the inclusion of a frugivore compensatory effect reduces the loss of recruitment resulting from the loss of muriquis to −47.2% instead of −54.0% in the scenario with all seed predators, to −36.2% instead of −49.5% in the scenario without peccaries and to –1.9% instead of –23.2% in the scenario without peccaries and agoutis (Fig. 2). Likewise, the inclusion of a compensatory effect reduces the cumulative loss of recruitment resulting from the loss of both muriquis and howler monkeys to −56.7% instead of −69.2% in the scenario with all seed predators, to −60.8% instead of −84.1% in the scenario without peccaries and to −46.7% instead of −82.4% in the scenario without peccaries and agoutis (Fig. 2). The loss of the largest-bodied seed disperser, the muriqui, had the highest impact on *C. mandioccana* recruitment success when the seed predator community remained intact. In contrast, the loss of howler monkeys and jacutingas – with the latter only in the compensation scenario – had the highest impact in the most defaunated area in terms of seed predators compared to more intact areas (Fig. 2).

## Discussion

We showed that the loss of large seed dispersers and predators synergistically decrease the recruitment success of a hardwood tree species. In defaunated scenarios, the antagonistic role of dispersers and predators will not result in a compensatory effect that is able to mitigate or reverse the limitations in the dispersal process, as previously suggested^17, 18^. In contrast, the combined effects of defaunation on large disperser and predator assemblages can exacerbate dispersal limitation and decrease recruitment success. However, the partial compensatory effect in the seed dispersal process mitigates the decrease in recruitment success. In addition, our model species still recruits without dispersers, suggesting that defaunation does not necessarily lead to the complete extinction of large-seeded species. A complete seed predator community and compensatory effects in the seed dispersal process buffer the negative effect of defaunation of large seed dispersers but are not sufficient to prevent a decrease in plant recruitment.

Partial compensation supports the hypothesis that processes carried out by a small number of key and functionally unique species, such as the dispersal of large seeds, are most sensitive to changes in biodiversity^17, 23^. The increasing contribution of howler monkeys and jacutingas to *C. mandioccana* seed dispersal when muriquis are absent indicates that smaller dispersers could benefit from the absence of large species, partially compensating for their role. Complete compensation or redundancy is achieved only when the seed dispersers are quantitatively and qualitatively similar^14, 19^, which is not what we found in our study system. The quantity component was never totally compensated. The quality of dispersal by howler monkeys and jacutingas was lower than that by muriquis in terms of seed germination success and dispersal distances, limiting their potential for compensation. When no clear distance effect is observed on seed/seedling survival, the functional attributes of the resilient seed disperser community compensate better (but not totally) the absence of muriquis because of the lack of long-distance seed dispersal benefits. However, the pattern of recruitment success is only one of the possible effects of defaunation: long-distance seed dispersal is a key process for plant populations, because it promotes gene flow and increases the probability of colonizing new habitats^24^. Therefore, high recruitment success observed in defaunated areas might hide a more pervasive effect: the strong reduction of gene flow due to the concentration of the seed rain under parent trees^25, 26^. It is thus urgent to combine field data of plant recruitment dynamics to plant population genetics in order to determine the possible evolutionary trajectories of these populations in the future^27^.

The contribution of each seed disperser is a context-dependent process, which is highlighted by the differential impacts of disperser extinction according to the seed predator community. This suggests that mechanisms other than compensation can influence the resilience of an ecosystem in response to a perturbation^17^. For example, we observed that the benefits of long distance seed dispersal provided by muriquis are almost eliminated when the seed predator community is disrupted. This effect mainly occurs because of the unexpected increase in seed predation at all distances, possibly due to the dominance of small rodents where large seed predators are absent. Indeed, our camera trap results show an up to 14-fold increase in the frequency of visits by small rodents in the seed predator-defaunated area compared to the area with an intact seed predator community. This is in line with recent evidence of a positive effect of defaunation on seed predation in Atlantic Forest explained by an increase in the population of small rodents due to the absence of large mammals (competitive release) and by a shift of some rodent species to a more peccary-like diet^28^. Therefore, the increase in seed predation and the absence of a distance effect in our seed predator-defaunated area might be due to the lack of satiation in the rodent population^29, 30^, inverting the expected plant recruitment trajectory based on the dispersal curves and confirming what has been observed for other plant species having peccaries and small rodents as their main seed predators^31^.

Our study shows the complexity of the cascading effects of defaunation on plant recruitment of one large-seeded species, highlighting compensatory effects and synergistic feedbacks, two ecological processes that are fundamental in exploring the effects of defaunation on the carbon stock ecosystem service. Indeed, a lack of information regarding these processes in future carbon stock modelling is likely to bias the estimate. Taking into account these complex cascading effects at the community level is challenging because of the difficulty to extrapolate the results of one plant species to the entire community since the response of each plant species depends on its traits and on the frugivore community with which it interacts. However, if enough knowledge is available, it is possible to predict defaunation effects from the study of the Janzen-Connell curves – dispersal and escape curves – as suggested by Terborgh^18^. The occurrence and magnitude of the effects of dispersal failure and compensation are directly linked to plant species traits. Seed size is likely to be related to the degree of redundancy in seed dispersal and predation networks^32^ while the capacity of plant species to germinate with pulp and recruit under parent trees could reduce the effects of dispersal failure^13^. Species like *C. mandioccana* that has the ability to germinate with pulp and rely on several seed dispersers might be more robust to changes in frugivore community. Therefore, defaunation effects on their recruitment dynamics should be mainly driven by establishment limitation. Defaunation effects on plant species with no redundancy of seed dispersers and unable to germinate with pulp or under parent trees^33^ should be driven by dispersal limitation since the seeds would not be able to establish even in absence of predation.

Future studies should thus attempt to identify patterns in the responses to defaunation across plant species traits to enable the inclusion of the magnitude of this variation when modelling the effects of seed disperser and predator loss. The inclusion of both mutualistic and antagonistic interactions is a necessary step to make more realistic predictions about the consequences of defaunation on ecosystem services. While it is clear that the extinction of large-seeded, animal-dispersed species results in a carbon stock loss that cannot be totally compensated for small-seeded or abiotically dispersed species^8–10^, the magnitude of the carbon loss may have been overestimated. There is a need to take into account the feedback induced by redundant frugivore and predator communities and the fact that many plant species can suffer from a decrease in recruitment rather than extinction^9^. It is also urgent to better understand the possible effect of density-dependent mortality after plant recruitment^34^. In our study, we identified the reduced recruitment success of undispersed seeds after one year. This higher density-dependent mortality is likely to affect later stages (e.g., the at least three-year recruitment stage in *Cryptocarya crassifolia* in Madagascar)^15^, and including this effect in future models would certainly improve our evaluation of carbon stocks.

Present-day seed dispersal, predation and post-dispersal events such as trampling and herbivory, have direct consequences on the future carbon stocks of tropical forests in a similar way to how past plant-animal interactions determined current carbon stocks. The ecological knowledge of the contribution of specific frugivore communities to plant recruitment allows to add value to their ecological services^35^. If an area is given a higher monetary value because it harbors a complete frugivore community, assuring the long-term maintenance of carbon stocks, policy makers and land owners should be encouraged to preserve both wildlife and forests, or even facilitate the restoration of extinct plant-animal interactions^36^. Estimates of the monetary value of ecosystem services are relatively common for pollination services but still extremely rare for seed dispersal^37^. Although one can argue that we cannot “value the priceless”, it should be noted that the objective is rather to increase the awareness of the general public and policy makers^37^ whose daily decisions are driven by the price that we explicitly or implicitly give to an ecosystem^38^. Consequently, bad decisions can be made because we have a better idea of the value of a plantation than the value of a forest^38^. To be able to do that, we need to better know the contribution of the frugivore community to forest regeneration. Actions to prevent charismatic animal extinction will contribute to ensuring the economic value of possible REDD+ programs (Reducing Emissions from Deforestation and forest Degradation). Based on the results of our studied species, a complete assemblage of seed dispersers and predators must be protected to guarantee REDD+ economic values but more studies are necessary to confirm this result for plant species with different seed traits. Since biomes with high carbon storage also harbor high biodiversity, the application of carbon-based conservation is likely to benefit many areas^39^. However, we must keep in mind that other conservation strategies must also be taken into account since carbon-poor regions with high biodiversity exist and might be jeopardized by the large-scale implementation of REDD+^39^. Although challenging and somewhat controversial, the attribution of monetary values to the ecological services provided by wildlife might be an important strategy to encourage their conservation.

## Methods

### Study site and model species

We studied the recruitment of a long-lived tree species, *Cryptocarya mandioccana* (Lauraceae), that relies on large mammals and birds to disperse its seeds^40^. *C. mandioccana* is a hardwood tree (0.72 g/cm^3^) that can reach up to 35 m in height and has yellow fleshy fruits containing one seed; the seeds are 1.34–3.00 cm in length and 1.16–1.92 cm in width^40, 41^. Their seeds are dispersed by two primate species (the southern muriqui, *Brachyteles arachnoides*, and the southern brown howler monkey, *Alouatta guariba*) and one large cracid bird (jacutinga, *Aburria jacutinga*)^40, 42^. Tapirs (*Tapirus terrestris*) are also thought to disperse *C. mandioccana* seeds but are likely not a reliable disperser for this species since our study did not identify seed dispersal events despite a quite large sampling effort (see Methods S4 in Supporting Information). Rodents (e.g, agoutis, *Dasyprocta* spp., pacas, *Cuniculus paca*, and small rodents such as *Euryoryzomys russatus*) and peccaries (white-lipped peccaries, *Tayassu pecari*, and collared peccaries, *Pecari tajacu*) are the main seed predators. Effective secondary seed dispersal by agoutis or other small rodents is quite unlikely since they rarely cache seeds smaller than 5 g^43^ (*C. mandioccana* seed mass = 2.4 g)^44^.

We worked in non-fragmented Brazilian Atlantic Forest to avoid any potential bias due to edge and fragmentation effects^45^. We studied the assemblage of seed dispersers and predators of *C. mandioccana* in three protected areas with distinct community compositions in 2011 and 2012 (Table 1; see Fig. S1 in Supporting Information). These protected areas used to contain all native seed dispersers and predators of *C. mandioccana* ^46^, but illegal hunting led to the severe population decline of large-bodied species, particularly muriquis, tapirs and white-lipped peccaries. The first site, located in the highlands of Carlos Botelho State Park (São Miguel Nucleus), harbors the complete set of seed dispersers (tapirs, muriquis, brown howler monkeys, and jacutingas) but lacks large seed predators (white-lipped peccaries)^47^; we classified it as “intact”. The second site, Ilha do Cardoso State Park, lacks tapirs and muriquis but harbors all seed predators (small rodents, agoutis, white-lipped and collared peccaries)^48^, we classified it as “moderately defaunated”. The third site, classified as “defaunated”, is located in the lowland forests of Carlos Botelho State Park (Sete Barras Nucleus) and harbors only jacutingas as seed dispersers, and small rodents and agoutis as seed predators^47^ (Table 1). All experiments were approved by the “Ministério do Meio Ambiente - MMA” and “Instituto Chico Mendes de Conservação da Biodiversidade” of Brazil through the authorization number 26261 and by the “Secretaria do Meio Ambiente” of Sao Paulo State through the authorization number 260108-000.577/2011. The study complies with current Brazilian laws.

### Data collection

We defined the recruitment success (*RS*_*s*,*m*_) of *C*. mandioccana at our three study sites as the percentage of seeds produced by a tree in one year that will result in seedlings surviving for one year^49^. We evaluated the *RS*_*s*,*m*_ by estimating the contribution of each disperser to dispersal, germination, and seedling establishment. The contribution of seed dispersers depends on the probability of seed removal (*P*_*s*_), the probability of germination after passing through the disperser’s gut (*G*_*s*_), the dispersal distance probability (*D*_*sm*_), and the seedling survival at each dispersal distance (*T*_*m*_), with *s* being the disperser and m the dispersal distance, modified from ref. 49.

1 $$R{S}_{s,m}=[{P}_{s}{G}_{s}\sum _{s=1\,}^{x}\sum _{m=1}^{y}({D}_{sm}{T}_{m})]\ast 100$$

We determined seed removal by arboreal frugivores through 108 to 172 h of focal observations of fruiting *C. mandioccana* trees in each area and by terrestrial frugivores through 270 to 463 days of camera trapping (see Supplementary Method S4 and Table S5). Seed germination success was assessed through *in situ* germination experiments of defecated seeds, seeds with pulp and seeds without pulp (see Supplementary Method S4). We determined the seed dispersal distances from conspecific trees by following habituated and semi-habituated groups of muriquis and howler monkeys, respectively, and by searching for tapir and jacutinga feces (see Supplementary Method S4). Finally, we assessed seedling survival through seed predation experiments at four distances from *C. mandioccana* trees (5, 15, 30, and 50 m) (see Supplementary Method S4).

### Data analyses

#### Contribution of seed disperser to recruitment success

We estimated the recruitment success and the contribution of each disperser using equation 1. The overall recruitment success corresponds to the activity of the current frugivore assemblage at each site, in 2011–2012, with data from the two years pooled together. We used a one-way ANOVA for a randomized block design to test the effect of seed treatment on germination success in each area. We used a generalized linear mixed model to test the effect of distance to the parent tree (fixed effect) on the one-year survival of dispersed seeds (response variable) using the “lme4” package^50^. As random effects, we included an intercept for trees as well as by-tree random slopes. The error structure of the response variable fits a Poisson distribution, and thus we used the logarithmic link function, and a χ² to test for significant effects of the explanatory variables in the model.

#### Compensatory effect and expected recruitment success

We simulated a sequence of seed disperser loss (from the largest to smallest frugivore) in the intact area to explore how seed disperser and predator extinctions affect *C. mandioccana* recruitment. We considered three scenarios based on the seed predator community and compensatory effects of the disperser community. The loss of seed dispersers without compensation consists of removing the contribution of the extinct disperser in the calculation of recruitment success (by zeroing out its seed removal probability and correcting the value of spat out and undispersed seeds in Table S2) without changing the values of the remaining dispersers. When a compensatory effect was added, the values of seed removal of the remaining dispersers as well as of spat out and undispersed seeds were changed according to field observations, i.e., data from the other communities. The effect of seed predators was calculated by applying the escape curves (survival according to distance) of the three study areas to the intact site.

### Data availability

The datasets generated during and/or analyzed during the current study are included in this published article (and its Supplementary Information files) or are available from the corresponding author on reasonable request.

## Electronic supplementary material

Supplementary Information S1 – S5
