# Supplementary material for: Synergistic effects of seed disperser and predator loss on recruitment success and long-term consequences for carbon stocks in tropical rainforests
Source: Sci Rep. 2017 Aug 9;7:7662. doi: 10.1038/s41598-017-08222-4 (PMC5550475; doi:10.1038/s41598-017-08222-4)
Supplement: Supplementary file 1 — Supplementary Information S1 – S5 [file 41598_2017_8222_MOESM1_ESM.pdf]

Synergistic effects of seed disperser and predator loss on recruitment success and long-term consequences for carbon stocks in tropical rainforests

Laurence Culot, Carolina Bello, João Luis Ferreira Batista, Hilton Thadeu Zarate do Couto, and Mauro Galetti

Supplementary Information S1 – S5:

Figure S1. Localization of the three study sites in the Atlantic Forest, state of São Paulo, Brazil

Table S2 Values of the parameters used to determine recruitment success of *C. mandioccana*

Figure S3 Dispersal, escape and recruitment curves of *C. mandioccana* in three different seed disperser and predator communities

Method S4 Additional details about the methodology

Table S5. Seed predation by terrestrial frugivores and granivores

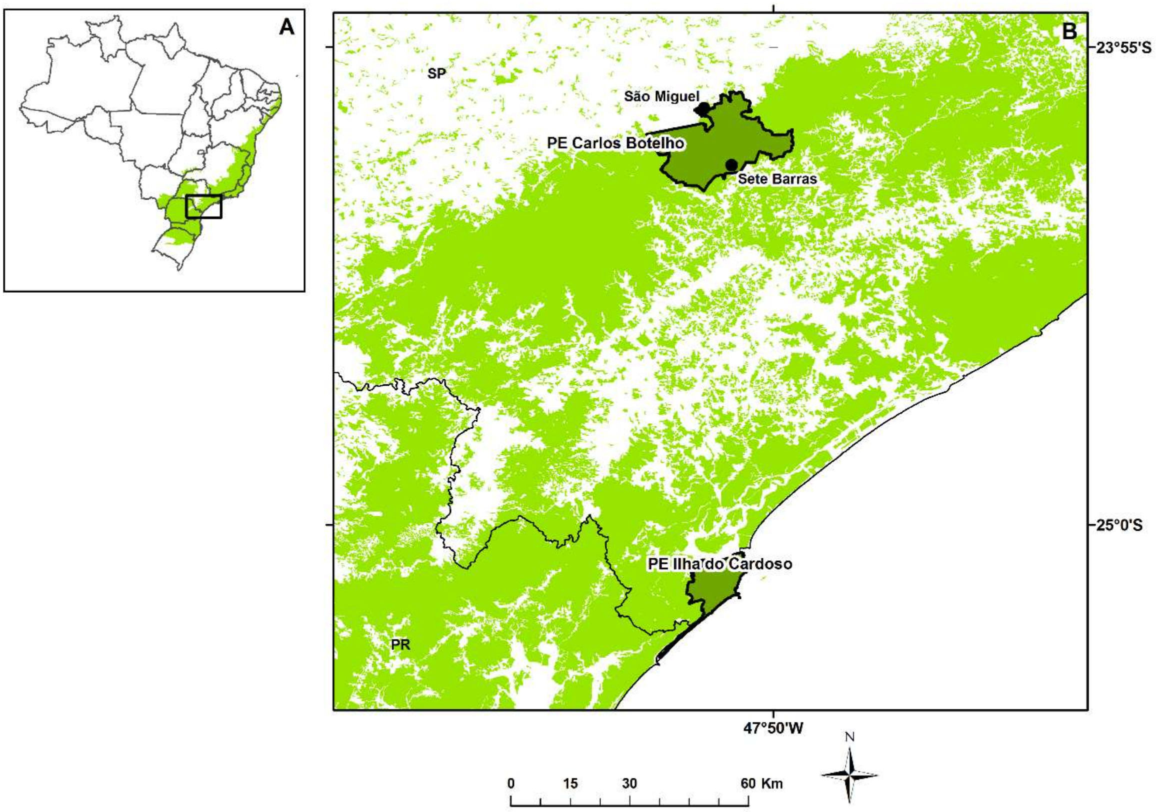

Supplementary Figure S1. Localization of the three study sites in the Atlantic Forest, state of São Paulo (SP), Brazil. (A) Map of Brazil showing the Atlantic Forest biome and the three study areas (SP: state of São Paulo, PR: Paraná). (B) São Miguel (highlands) and Sete Barras (lowlands) in the Carlos Botelho State Park (PE Carlos Botelho = Parque Estadual Carlos Botelho), and the Ilha do Cardoso State Park (PE Ilha do Cardoso = Parque Estadual Ilha do Cardoso). The map was created using ArcGis 10.3 software (http://www.esri.com/software/arcgis/arcgis-for-desktop).

Supplementary Table S2. Values of the parameters used to determine recruitment success of *C. mandioccana*. Recruitment success (RS_{sm}) of *C. mandioccana* (2011-2012) calculated from the parameters included in equation 1 for the communities in the highlands of the Carlos Botelho State Park (CB-High), the Ilha do Cardoso State Park (IC), and the lowlands of the Carlos Botelho State Park (CB-Low). CB-High harbors muriquis (mur), howler monkeys (how), and jacutingas (ja) as seed dispersers and small rodents as seed predators. IC harbors howler monkeys and jacutingas as seed dispersers and small rodents, agoutis, and peccaries as seed predators. CB-Low harbors jacutingas as seed dispersers and small rodents and agoutis as seed predators. The percentage contribution (% contrib) to recruitment success was calculated for undispersed (undisp), spat out (spat), and swallowed (and defecated) seeds (swal) by each of the dispersers (Disp); undet: undetermined seed disperser (muriqui, howler monkey or jacutinga).

Com	Seed state	Disp *s*	Disp dist *m* (in m)	P_s	G_s	D_{sm}	T_m	Contrib	% contrib	RS_{sm} (%)
**CB-High**	Undisp	none	0	0.06	0.36	1	0.04	0.0008	0.93	9.02
	spat	undet	0	0.09	0.52	1	0.04	0.002	2.26	
	swal	mur	0-5	0.41	1	0	0.04	0	29.99	
			6-15	0.41	1	0.14	0.17	0.0099		
			16-30	0.41	1	0.17	0.02	0.0017		
			30 and more	0.41	1	0.68	0.05	0.0154		
	swal	how	0-5	0.41	0.96	0.14	0.04	0.0023	66.14	
			6-15	0.41	0.96	0.84	0.17	0.0572		
			16-30	0.41	0.96	0.02	0.02	0.0002		
			30 and more	0.41	0.96	0	0.05	0		
	swal	ja	0-5	0.01	0.77	0.23	0.04	0	0.69	
			6-15	0.01	0.77	0.64	0.17	0.0006		
			16-30	0.01	0.77	0.1	0.02	0		
			30 and more	0.01	0.77	0.02	0.05	0		
**IC**	undisp	none	0	0.19	0.54	1	0.24	0.0243	15.66	15.51
	spat	undet	0	0.09	0.52	1	0.24	0.0114	7.38	
	swal	how	0-5	0.47	0.96	0.14	0.24	0.015	58.05	
			6-15	0.47	0.96	0.84	0.19	0.0719		
			16-30	0.47	0.96	0.02	0.43	0.0031		
			30 and more	0.47	0.96	0	0.54	0		
	swal	ja	0-5	0.16	0.77	0.23	0.24	0.007	18.91	
			6-15	0.16	0.77	0.64	0.19	0.0155		
			16-30	0.16	0.77	0.1	0.43	0.0054		
			30 and more	0.16	0.77	0.02	0.54	0.0014		
**CB-Low**	undisp	none	0	0.47	0.84	1	0.11	0.0432	33.81	12.77
	spat	undet	0	0.07	0.8	1	0.11	0.0061	4.79	
	swal	ja	0-5	0.41	0.77	0.23	0.11	0.008	61.40	

6-15	0.41	0.77	0.64	0.30	0.0603
16-30	0.41	0.77	0.1	0.24	0.0076
30 and more	0.41	0.77	0.02	0.40	0.0025

Ps: probability of removal
Gs: probability of germination
Dsm: Probability of dispersing the seed at distance m
Tm: one-year survival of seedling dispersed by disperser s at distance m
Contrib: Contribution of the disperser to total recruitment success (sum of the product of Ps, Gs, Dsm, and Tm of undispersed, spat out, and swallowed seeds of each disperser separately)

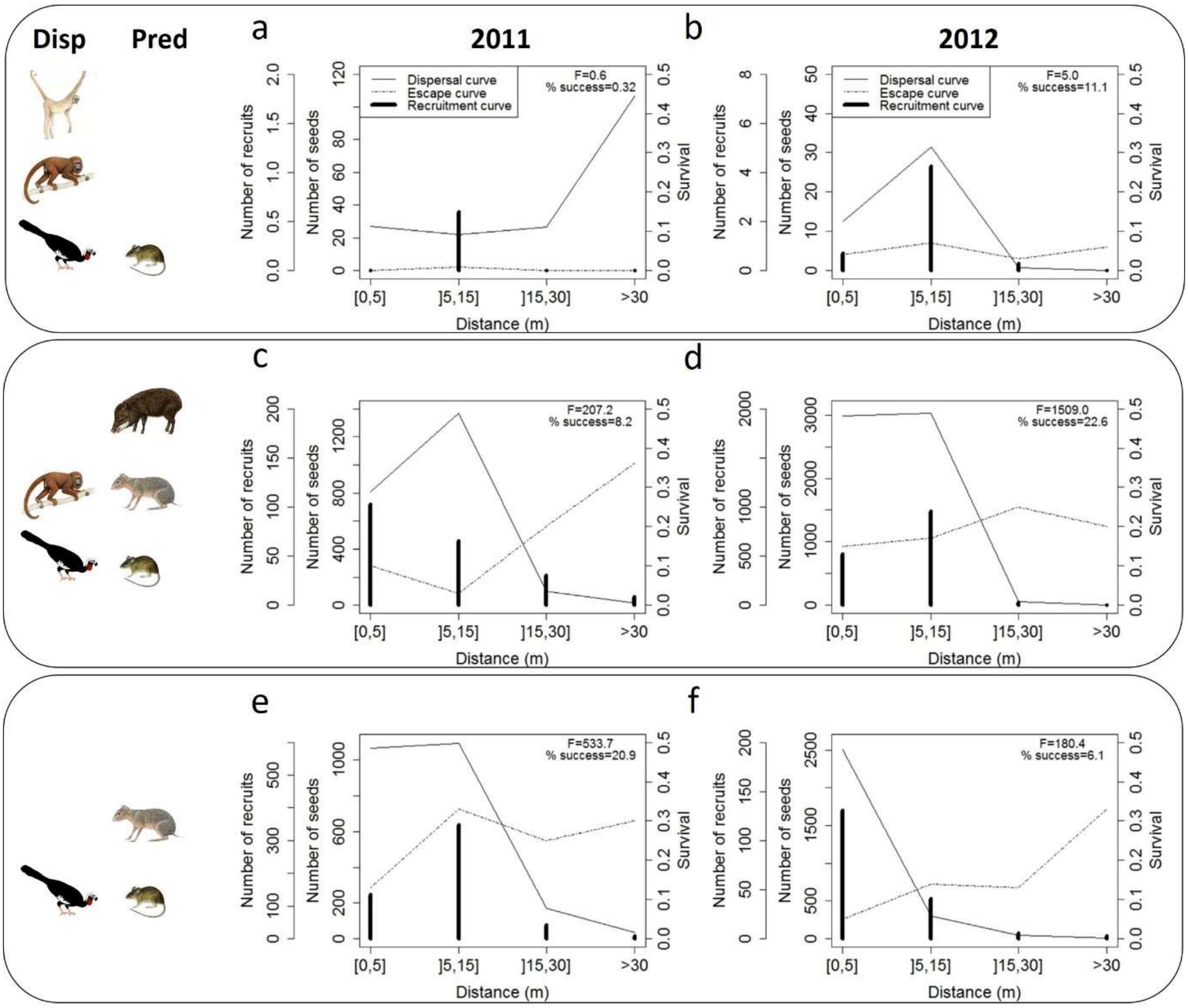

Supplementary Figure S3. Dispersal, escape and recruitment curves of *C. mandioccana* in three different seed disperser and predator communities. The communities of (a, b) the Carlos Botelho State Park – Highlands, (c, d) the Cardoso Island State Park, and (e, f) the Carlos Botelho State Park – Lowlands, sampled in 2011 and 2012, differ in their seed disperser (Disp) and seed predator (Pred) communities. The number of one-year seedlings produced per tree and per year (F), and the percentage recruitment success (% success), based on seed production of *C. mandioccana* trees, is given for each area and each year. The dispersal curves combine the percentage of seed removal, the frequency of dispersal distances of the current dispersers of the community, and the mean seed production of *C. mandioccana* trees, showing the number of seeds dispersed at each distance category. The escape curves show the survival probability of seeds without pulp and the recruitment bars represent the number of recruits (1-year seedlings) per year, per tree and per distance category. (Illustrations of muriqui and howler monkey - Copyright Stephen D. Nash; peccary, agouti, and rodent – Copyright Fiona A. Reid; jacutinga – Copyright Fabio Martins Labecca; authorized by the authors).

Supplementary method S4. Additional details about the methodology

Seed production: We placed four to seven 1 m^2 seed traps, representing at least 6 to 10% of the tree crown area, under six *C. mandioccana* fruiting trees in each site during two fruiting seasons (2011 and 2012). Seed traps were made of a nylon mesh bag (1 mm mesh) supported 1m above the ground by four PVC tubes forming a horizontal square. Once a month, we counted and removed the fruits and seeds fallen in the traps distinguishing totally or partly eaten fruits, spat out seeds, preyed seeds (in pieces), and defecated seeds. We estimated seed production by dividing the total number of seeds fallen in the traps by the number of traps and multiplying the result by the tree crown area. We did not include the defecated seeds because they might have come from other trees. Since *C. mandioccana* fruits have no true husk left behind by seed dispersers, seed traps did not sample the seeds swallowed by frugivores. In order to correct this bias, we estimated the number of seeds swallowed per tree by each arboreal frugivore in each site and added it to the result. This estimation was based on focal-observation data that provided the ratio of swallowed seeds per spat out seeds for each frugivore in each study site (see the "removal probability" section). Using the number of spat out seeds and this ratio, we could calculate the total number of seeds swallowed by arboreal frugivores for each tree. This data was used in the calculation of the removal probability.

Removal probability (P_s): We estimated seed removal by arboreal and terrestrial frugivores separately. We estimated seed removal by arboreal frugivores through focal observations of 3-5 *C. mandioccana* fruiting trees per study site from 6.00 am to 5.30 pm for a total of 172h in São Miguel (84h in 2011 and 88h in 2012), 108h in the Cardoso Island (43h in 2011 and 65h in 2012), and 153h in Sete Barras (103h in 2011 and 50h in 2012). We registered the visiting species, the times of arrival and departure, the duration of feeding and resting behaviors, and the number of seeds swallowed and rejected. When a group of animals belonging to the same species arrived and fed at a focal tree at the same time, we collected data on one focal individual and noted the times of arrival and departure of the others. Combining focal observation with seed production data, we estimated for each study site the mean percentage of preyed seeds (pre-dispersal predation), undispersed seeds (seeds with pulp), spat out seeds (seeds without pulp), and swallowed seeds. These estimates were necessary to calculate the final recruitment success of *C. mandioccana*.

To estimate seed removal by terrestrial frugivores under parent trees, we installed Bushnell camera traps under *C. mandioccana* trees, where we gathered 50 fruits close to the camera, allowing the identification of the frugivores removing the seeds. We totalized 270 camera-days in São Miguel (167 in 2011 and 103 in 2012), 463 in the Cardoso Island (104 in 2011 and 359 in 2012), and 370 in Sete Barras (55 in 2011 and 315 in 2012). We also randomly checked tapir latrines (N = 28) for the presence of *C. mandioccana* seeds. These data did not reveal any seed dispersal event by terrestrial frugivores but enabled the identification of the main seed predators of *C. mandioccana* (Table S5).

Germination probability (G_s): In order to take into account the possible environmental heterogeneity in each area (e.g: distance to river, density of C. *mandioccana* trees) for the evaluation of germination success, we set the germination experiments according to a

randomized block design in which each block contained three treatments. We set up ten blocks per area (except in Sete Barras where we set up 9 blocks because of the low availability of seeds for the experiment), consisting of metallic cages of 50 x 50 x 25 cm with 1 cm^2-wire mesh, spaced by 50m, along one transect. Each block contained one replicate of each treatment. Each treatment contained five seeds. The treatments were the following: 1) seeds with pulp, 2) seeds whose pulp had been removed manually, and 3) seeds defecated by either jacutingas, howler monkeys or muriquis (depending on their presence in each site). We checked for seed germination once a week for 21 weeks, which corresponds to the time when we had not observed any new germination event in the previous 4 weeks. Germination was assessed by the emergence of radicle. Through hemispherical pictures and analysis in Hemiview software [1] we characterized the light environment at random points in the three study sites and in defecation sites. We verified that the light environment was suitable for seedling establishment of *C. mandioccana* in more than 85% of cases (see the "Assessment of light requirements" section). Therefore, we only controlled this factor in the germination experiment by avoiding placing the experimental block in extreme light conditions (very high or very low ground cover).

Dispersal distances (D_{sm}): We searched for *C. mandioccana* seeds in feces of muriquis and howler monkeys while following habituated and semi-habituated groups in 2011. We searched for seeds dispersed by tapirs and jacutingas by randomly looking for latrines and defecations, respectively. Every dispersed seed and the nearest *C. mandioccana* adult trees were mapped. We estimated the dispersal distance using ArcGis 10 software.

Seedling survival (T_m): We determined the predation and survival of seeds without pulp using seed predation experiments around 12 *C. mandioccana* fruiting trees per site. We deposited seeds without pulp at 5, 15, 30, and 50 m from the parent tree to test for a possible distance effect. We checked the seeds after 12 months for survival and seedling establishment. We repeated the experiment in 2011 and 2012. Since the seedling survival experiments was done using seeds without pulp (instead of defecated seeds), the results actually represent the product of germination and survival probabilities, i.e the seed-seedling transition probability. Therefore, for the calculation of the recruitment success, we estimated a survival probability at each distance in each study area by dividing the seed-seedling transition probability by the germination probability of seeds without pulp. Using equation 1, we multiplied each disperser-specific germination probability by the distance-specific survival probability in each area.

Assessment of light requirements of *C. mandioccana*: In evergreen forests, the leaf-filtered daylight is one of the most important factors affecting seed germination [2]. Light may serve as a resource but also as a signal. Light conditions may vary in spectral composition, in photon flux density and in duration of exposure. Several factors such as soil or leaf canopy density can affect light conditions. The leaf canopy reduces the photon flux density of all wavelengths – but much more in the photosynthetically active part – that arrive on seeds. Seeds are able to detect shading by established plants and can respond by delaying their germination [2].

We characterized the light environment at 50 random points and above 35-38 *C. mandioccana* seedlings in each study site through the analysis of hemispherical pictures in the Hemiview canopy analysis software version 2.1 Delta-T [1]. The pictures were taken 30-50 cm above ground level using a horizontally leveled digital camera (Coolpix 5700, Nikon, Tokyo, Japan) orientated to the north and aiming at the zenith using a fisheye lens of 180° field of view (FCE9 0.2x). The comparison of light environments between seedlings and random points can reveal possible light environment restrictions for seedling establishment of *C. mandioccana*. The second step consisted in taking hemispherical pictures using the same protocol above the defecation sites of muriquis, howler monkeys, and jacutingas where *C. mandioccana* seeds were deposited in order to determine if the light environment at deposition sites is suitable for *C. mandioccana* seedling establishment.

We used a discriminant analysis to select, among six common indexes of solar radiation and canopy structure given by the Hemiview software, those allowing the discrimination between "suitable" and 'unsuitable" sites for *C. mandioccana* seedling establishment.

Out of six indexes of solar radiation and canopy structure, the discriminant analysis kept two (Lambda Wilk = 0.76, $P < 0.0001$): the proportion of indirect light (ISF: Lambda Wilk = 0.10, $P < 0.000001$) and the effective ground covered by canopy (GndCover: Lambda Wilk = 0.86, $P < 0.000001$). *C. mandioccana* seedlings did not show any strong preference for a specific light environment since their ISF and GndCover landscape mostly overlapped those of random points (Fig. 1). More than 85.3% (122/143) of random points were categorized as "suitable" for *C. mandioccana* seedling establishment by the discriminant analysis, showing the potential high availability of recruitment sites in the three areas. Only extreme light environments seem unsuitable for *C. mandioccana* seedling establishment: high GndCover values combined with low ISF values or low GndCover values combined with high ISF values.

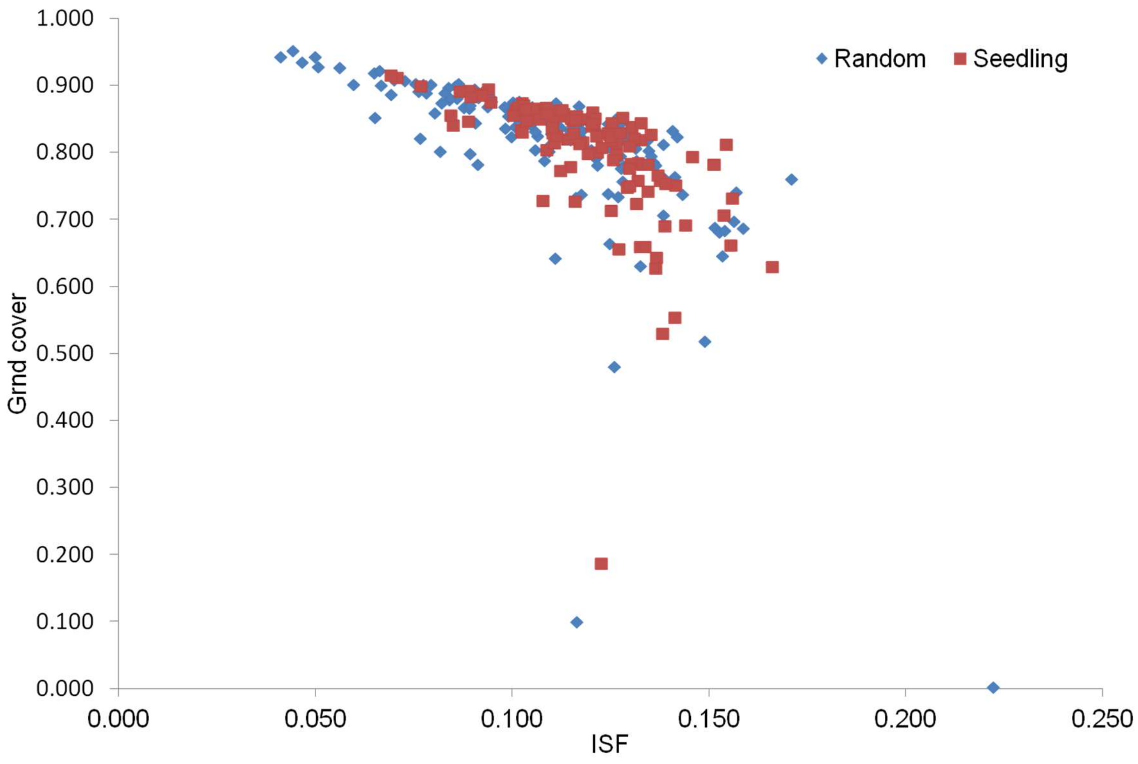

Fig. 1 of Supplementary method. Light environment of random points and above seedlings in the three study areas described as the proportion of indirect light (ISF) and the effective ground covered by canopy (Gnd cover).

Since *C. mandioccana* did not show any strong restrictions regarding the light environment, most of seed deposition sites induced by the three main dispersers were categorized as "suitable" for seedling establishment: 98.4% of muriquis' defecation sites (62/63), 85% of howler monkeys' defecation sites (17/20), and 100% of jacutingas' defecation sites (11/11) (Fig. 2).

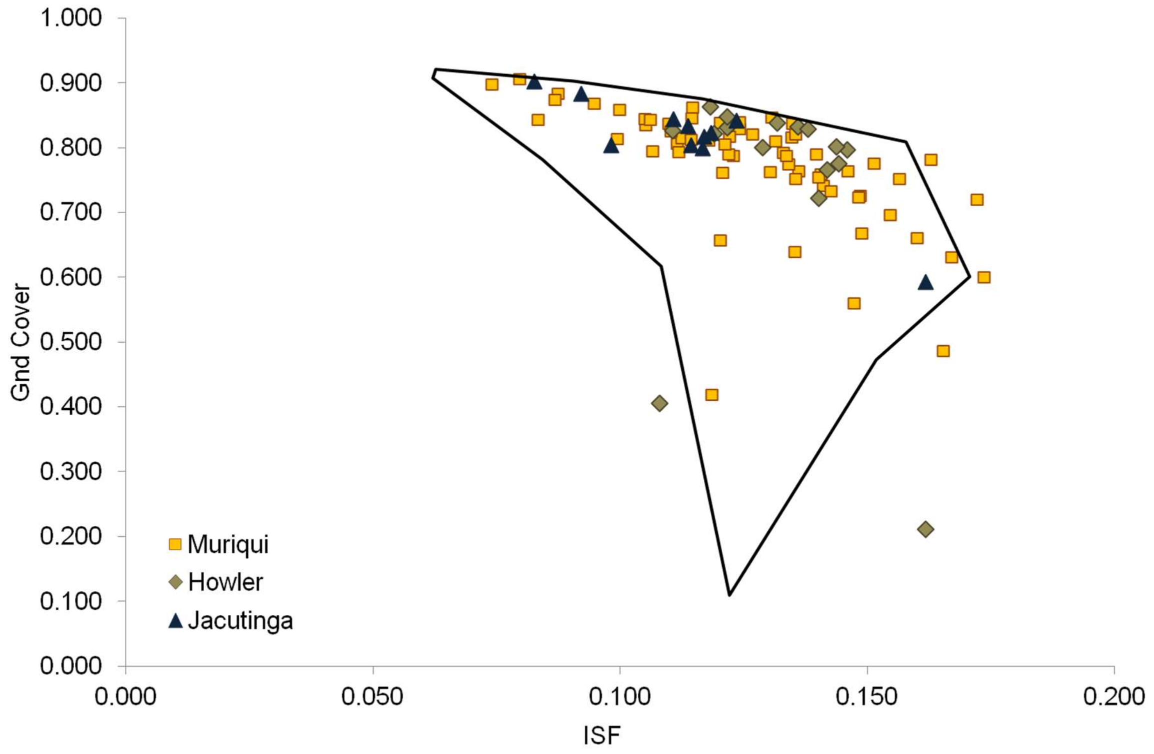

Fig. 2 of Supplementary method. Light environment of muriquis', howler monkeys', and jacutingas' defecation sites described as the proportion of indirect light (ISF) and the effective ground covered by the canopy (Gnd cover). The black polygon represents the light environment area of seedlings and suitable random points.

References

1 Delta-T-Devices. HemiView canopy analysis software Version 2.1. *Delta-T Devices Ltd., Cambridge* (1998).

2 Pons, T. L. Seed responses to light in *Seeds: the ecology of regeneration in plant communities* (ed M Fenner) 237-260 (CAB International, 2000).

Supplementary Table S5. Seed predation by terrestrial frugivores and granivores. Mammal species registered by camera trapping with *C. mandioccana* fruits as bait in the highland forest of the Carlos Botelho State Park (CB-High), the Cardoso Island (IC), and the lowland forest of the Carlos Botelho State Park (CB-Low) in 2011 and 2012. A total of 50 *C. mandioccana* fruits were placed in front of a camera trap for 1.5 month, in two different places in CB-High and CB-Low and four different places in IC. We registered whether the species only passed by but did not prey the seeds (No), preyed the seeds (Yes) or seemed to feed on fruits but without the certainty that they preyed the seeds (Yes?).

	**Total camera trap days**		**% seeds eaten after 1.5 mo**			**Freq of visits**		**Preyed upon seeds?**
**Site**	**2011**	**2012**	**2011**	**2012**	**Species**	**2011**	**2012**	
CB-High	167	103	37.8	44.1	Small rodents	0.7	0.2	Yes
					Nasua nasua	0.01	0	Yes?
					Cuniculus paca	0	0.01	Yes?
					Tapirus terrestris	0.006	0	No
					Eira barbara	0.006	0	No
IC	104	359	11.3	1.3	Small rodents	0.11	0.05	Yes
					Dasyprocta azarae	0.11	0	Yes
					Didielphis aurita	0.06	0.003	No
					Tayassu pecari	0.05	0.008	Yes
					Pecari tajacu	0	0.002	Yes
CB-Low	55	315	24	10.3	Small rodents	0.02	0.15	Yes
					Dasyprocta azarae	0.04	0.1	Yes
					Didelphis aurita	0.04	0.07	No
					Mazama sp.	0.05	0.01	Yes
					Cuniculus paca	0	0.03	Yes
					Pecari tajacu	0	0.01	Yes
					Tapirus terrestris	0	0.006	No
